# Innovative Molecular Target and Therapeutic Approaches in Nonalcoholic Fatty Liver Disease/Nonalcoholic Steatohepatitis (NAFLD/NASH) 2.0

**DOI:** 10.3390/ijms23147894

**Published:** 2022-07-18

**Authors:** Mariapia Vairetti, Giuseppe Colucci, Andrea Ferrigno

**Affiliations:** 1 Department of Internal Medicine and Therapeutics, University of Pavia, 27100 Pavia, Italy; andrea.ferrigno@unipv.it; 2 Division of Gastroenterology and Hepatology, Department of Pathophysiology and Transplantation, Fondazione IRCCS Ca’ Granda Ospedale Maggiore Policlinico, University of Milan, 20122 Milano, Italy; giuseppe.colucci@guest.unimi.it

Nonalcoholic fatty liver disease (NAFLD) is among the most common liver diseases worldwide, affecting up to 20–30% of the human population [1]. NAFLD is usually associated with metabolic syndrome, which is characterized by increased abdominal fat, insulin resistance, high blood pressure and hyperlipidemia. In about 10% of individuals, NAFLD progresses to steatohepatitis (NASH) and, through fibrosis, to cirrhosis with the potential risk of developing hepatocellular carcinoma (HCC). The latter is among the most important causes of liver transplantation in the US with consequent relevant social and economic impact [2]. Nonetheless, no drug has been registered with the specific indication of NASH, leaving important medical needs still to be met.

The main intent of this Special Issue is to highlight new pathogenic mechanisms that contribute to the progression of NAFLD and to indicate innovative therapeutic targets/molecules.

Experimental models used for the characterization of NAFLD pathogenesis present strengths and weaknesses with regard to their comparability to the human disease. According to recent reports, the methionine and choline-deficient (MCD) diet remains one of the most effective in inducing NAFLD/NASH [3]. Innovative experimental models, such as the use of adult medaka strain, administered with Gubra-Amylin nonalcoholic steatohepatitis (GAN), have been proposed as an efficient fatty liver model [4].

On the other hand, non-invasive methods to diagnose and stage NAFLD/NASH are required. Wegrzyniak et al. summarize and discuss the current status of the development of innovative imaging markers for processes involved in liver fibrogenesis, including immune cells, activated fibroblasts and collagen deposition [5].

Many pathophysiological mechanisms are involved in the development of NAFLD and its progression to NASH, such as: oxidative stress, lipotoxicity, intestinal microbiota dysbiosis, endocrine alterations, adipokine dysregulation, and endoplasmic reticulum stress; lipotoxicity is discussed as a leading cause of NASH in the review by Branković et al. [6]. Lipid metabolism is affected by gut microbiota, and dysbiosis, which occurs in the development of NAFLD, stimulates both lipid accumulation in the liver and lipotoxicity. In detail, abnormalities in the intestinal environment, such as the gut microbiota, metabolites, and immune system have been demonstrated in NAFLD [7]. The documented increased intestinal permeability results in hepatic macrophage activation induced by the leakage of antigens, endotoxins, and other proinflammatory substances into the bloodstream. Up-to-date evidence suggests the presence of increased gut permeability in patients with NAFLD, helping in identifying patients who may benefit from intestinal barrier interventions such as the use of probiotics, prebiotics and postbiotics [7,8].

The review of Yamaguchi et al. summarizes the molecular mechanisms of HCC development focusing on the TGF-beta/smad signaling pathway, implicated in hepatic carcinogenesis and fibrogenesis [9].

The crucial role of specific molecules and their mediated signaling pathways in NAFLD and NASH is summarized by Zhang and Yang [10]. In detail, the peroxisome proliferator-activated receptors (PPARs), nuclear receptor proteins, have been shown to play a vital role in modulating fatty acid and glucose metabolism. The same is true for Krüppel-like factors (KLFs): in KLF10-deficient mice, a high-sucrose diet promoted the progression of hepatic steatosis, inflammation, and fibrosis. Insulin resistance (IR), a component of metabolic syndrome, contributes to the development of NAFLD and NASH. Moreover, in relation to the Wnt signaling pathways, the expression of Wnt5a and Wnt11 increased by 3-fold and 15-fold, respectively, in the diet-induced mouse NASH model, indicating the involvement of non-canonical Wnt signaling in NASH progression [10].

Another signaling pathway is the tumor suppressor gene p53, which plays an important role in the pathogenesis of NAFLD and NASH as well as the Vascular Cell Adhesion Molecule 1(VCAM-1); this is significantly increased in murine and human NASH, mediating the migration of inflammatory cells and resulting in the progression of NASH [10].

With new data rapidly emerging on the complex pathogenesis of NASH, new therapeutic approaches are being developed, focusing on hepatic steatosis, inflammation, and fibrosis. Innovative therapeutic approaches in the treatment of NAFLD/NASH target molecules involved in its pathophysiological pathway, including PPARs agonists, glucagon-like peptide-1 (GLP-1) agonists, sodium/glucose transport protein 2 (SGLT2) inhibitors, farnesoid X receptor (FXR) agonists, probiotics, and symbiotics, as reviewed by Filipovic et al. [11]. In addition, integrins have been proposed as new therapeutic targets for hepatic fibrosis. They do not act prominently in fibrogenesis but are closely related to or regulate matrix proteins, fibroblasts, and TGFβ. Indeed, the role of integrins expressed in activated fibroblasts/HSCs, α8β1, and α11β1 as ideal therapeutic targets in fibrosis, as reviewed by Yokosaki and Nishimichi [12].

Some drugs have been already tested in clinical trials in the therapy of NAFLD, including molecules used to treat type 2 diabetes mellitus (T2DM), one of the main risk factors for NAFLD. Among all the drugs used in T2DM therapy, the sodium-glucose cotransporter (SGLT2) inhibitors seem to be more promising in patients with NAFLD. Indeed, in several trials, these compounds appear to reduce liver fat content, AST/ALT levels, and even liver stiffness, suggesting they may be effective in future NAFLD-specific treatment protocols [13]. In particular, the treatment with empagliflozin, an SGLT-2 inhibitor, not only decreases hepatic lipid accumulation in diet-induced NAFLD [14] but in prediabetic rats, it mitigates hepatic steatosis and modulates the expression of genes involved in lipogenesis and lipid storage [15].

Hepatoprotective compounds of natural origin are promising therapeutic tools in counteracting the pathophysiological alterations caused by an excess of free fatty acids (FFAs) in the hepatocytes. Thus, the study of food bioactive compounds became an attractive approach to identifying molecules with beneficial properties in NAFLD. A review of Salvoza et al. provides details about the protective effects of compounds such as coffee, tormentic acid, verbascoside and silymarin and their mechanism of action in ameliorating the critical pathological events involved in NAFLD [16]. In addition, the same authors showed, in an experimental model of NAFLD, that APPLIVER (Cydonia oblonga cell extract, expressed in triterpenic acid) and ACTEOS (Lippia citriodora cell extract, expressed in acteoside) exert hepatoprotective effects [17]. In detail, ACTEOS reduced both the TNF-α and ROS production and attenuated collagen deposition. APPLIVER also showed inhibition of both TNF-α production and collagen deposition caused by FFA accumulation [17].

An additional attempt to prevent the development of hepatic steatosis by the use of dietary supplements is reported by the paper of Arulkumar et al. These authors found that soyasapogenol C, from fermented soybean (Glycine Max), by means of inducing the phosphorylation of AMP-activated protein kinase (AMPK), stimulates the nuclear translocation of PPARα, so inhibiting triglyceride accumulation [18].

Furthermore, pyrroloquinoline quinone (PQQ), a potent dietary antioxidant, has been found to control maternal obesity that significantly elevates the risk of pediatric NAFLD: PQQ supplementation during gestation and lactation stimulates pathways involved in the biosynthesis of long-chain fatty acids and plays a role in modifying specific bioactive lipid critical for protecting against NAFLD risk in later life [19].

Using 5-aminolevulinic acid (5-ALA), a precursor in the heme biosynthetic pathway, Hashimoto et al. showed, in palmitate-induced ER stress and lipoapoptosis, an attenuation of Glucose-Regulated Protein 78 (GRP78) expression and hepatocyte lipoapoptosis via heme oxygenase-1 (HO-1) induction [20].

As thyroid hormone receptor β (THRβ) activation has shown beneficial effects on metabolic alterations, an attempt to reduce steatosis was performed using TG68, a novel THRβ agonist. This drug strongly reduced hepatic fat accumulation and liver injury in mice fed a high-fat diet (HFD) with no detectable toxicity in extra-hepatic tissues, such as the kidney or heart [21].

Another attractive candidate for the treatment of NASH is Losartan (Cozaar), an angiotensin II receptor antagonist that has been known to attenuate the progression of NASH in animal models of obesity. Cheng-Hui Wang et al. demonstrated, in a murine model of NAFLD, that Losartan prevents hepatic steatosis and macrophage polarization by inhibiting hypoxia-inducible factor-1α (HIF-1α) [22].

The complex pathogesis of NAFLD/NASH, its worldwide prevalence and the lack of specific treatments justifies the ever-increasing effort in the development of new diagnostic tools and new specific therapies. This Special Issue intends to provide an update on the state of the art of the management of NAFLD/NASH.
